# Exploring the combined effect of hypertension and hypoxic burden index on cognition and underlying sex differences

**DOI:** 10.1002/alz70856_100148

**Published:** 2025-12-25

**Authors:** Jiefei Yu, Aaron Kin Fu Lam, Shawn Dexiao Kong, Sharon L. Naismith

**Affiliations:** ^1^ The University of Sydney, Camperdown, NSW, Australia; ^2^ Healthy Brain Ageing Program, Brain and Mind Centre, University of Sydney, Camperdown, NSW, Australia; ^3^ Healthy Brain Ageing Program, Brain and Mind Centre, School of Psychology, Faculty of Science, University of Sydney, Australia, Camperdown, NSW, Australia

## Abstract

**Background:**

Hypertension is a known modifiable risk factor for dementia. Evidence shows that obstructive sleep apnea may affect both hypertension and cognition. However, the combined effect of hypertension and obstructive sleep apnea on cognition remains unexplored. It is also unknown whether sex differences might modify this relationship. Therefore, we aimed to investigate hypertension, obstructive sleep apnea and their combined effect on cognition, as well as any sex differences underpinning.

**Method:**

This study included 114 adults over the age of 50 (67% females) without dementia. Systolic blood pressure was collected on the night and morning of a sleep study to derive hypertension status, whereas the hypoxic burden index was measured during sleep to assess the severity of obstructive sleep apnea. Comprehensive neuropsychological assessments were used to evaluate cognition outcome. The analysis of covariance (ANCOVA) was used in this study.

**Result:**

Individuals with hypertension had worse executive function (*p* = 0.031, η^2^=0.079) than those with normotensive. Hypoxic burden index or its combined effect with hypertension did not have effects on any cognitive domains (*p* >0.05). In the male group, the hypertensive group had worse executive function (*p* = 0.011, η^2^=0.401) and processing speed (*p* = 0.034, η^2^=0.267), compared to the normotensive group.

**Conclusion:**

Our study showed that hypertension is linked to executive dysfunction, and this effect seems to be driven by males. Furthermore, the hypoxic burden index was not a significant predictor of cognitive outcomes in our cohort, highlighting the need to explore other metrics to study the effects of obstructive sleep apnea on cognition.

